# Extremely low effective impedance in stratified graphene-dielectric metamaterials

**DOI:** 10.1038/s41598-022-15841-z

**Published:** 2022-07-08

**Authors:** Ruey-Bing Hwang

**Affiliations:** Institute of Communications Engineering, College of Electrical and Computer Engineering, National Yang Ming Chiao Tung University, Hsinchu, 30050 Taiwan

**Keywords:** Metamaterials, Computational science

## Abstract

The periodic reflections in frequency were observed in a stack of graphene layers and generally reported as a series of mini photonic bandgaps owing to the multiple interference by the graphene layers. In this research, the Floquet-Bloch theory was employed to obtain the effective refractive index and Bloch impedance for understanding the wave propagation characteristic therein. Interestingly, the periodic reflections were found to occur in the frequency band having drastic variation in complex Bloch impedance and effective refractive index as well, wherein a Floquet-Bloch mode having pure real effective refractive index and extremely low Bloch impedance exists.

## Introduction

Graphene is believed to be one of the most striking materials with its optical property defined only by the fundamental constants rather than on material parameters. The scattering characteristics of a suspended graphene is determined merely by the fine structure constant associated with quantum electrodynamics^1^. Experimental studies indicated that the optical sheet conductance of graphite per graphene layer is very close to the theoretically expected value of dynamical conductance of isolated monolayer graphene^2,3^. Moreover, for few graphene layers ($<6$), the structure behaves as a superposition of single sheet acting as independent two-dimensional electron gases; the absorptance is proportional to the number of layers^4^. The full expression for the optical conductivity based on the general noninteracting tight-binding model was developed for the scattering analysis of a graphene layer^5^. Having the closed-form expression for graphene optical conductivity, numerical electromagnetic field simulation for the structure consisting of single or multilayered graphene becomes more realistic and sophisticated^6,7^. Due to the reconfiguration of graphene optical conductivity by electrically or magnetically tuning the Fermi level (chemical potential) of a graphene sheet, some potential applications were proposed and implemented; to mention a few, a waveguide-integrated electroabsorption modulator based on monolayer graphene was developed by electrically tuning the Fermi level of the graphene sheet^8^. The Faraday rotation turning the polarization by several degrees through a single- and multilayered graphene was demonstrated in modest magnetic fields^9^. By stacking graphene bearing quartz substrate on a ground plane, an optically transparent broadband absorbers operating in millimeter wave region was achieved^10^. A ultra-broadband absorber made of multilayered graphene metamaterial able to absorb $90\%$ of the incident wave under normal incidence in the frequency range of 1.12–3.78 THz was reported^11^. A metamaterial consisting of weakly absorbing alternating graphene layers separated by lossless dielectric was fabricated to serve as a polarization-independent extremely broadband absorber covering almost the entire solar spectrum over a large angular range^12^. A graphene-based tunable hyperbolic metamaterials was designed for enhanced absorption in far-infrared frequencies^13^. The tunable propagation properties of 3D Dirac semimetal patterned metamaterial structures was symmetrically investigated in the terahertz regime^14^. The propagation properties of all-dielectric metamaterials based on a $SiO_2$-*Si* asymmetric hybrid block, including the effects of structural parameters, asymmetrical degrees, carrier doping concentrations, and graphene Fermi levels were reported^15^. Tunable terahertz Dirac-semimetal hybrid plasmonic waveguides was systematically investigated^16^. Moreover, 3D Dirac semimetal supported tunable TE modes was researched^17^. Concerning the fabrication technology development, multilayered metamaterial consisting of alternating monolayer graphene oxide/graphene and dielectric layers without a transfer step was successfully developed^18^.

Regarding the scattering characteristics of a 1D metamaterial made of a stratified graphene-dielectric structure, the transfer matrix method was popularly employed to obtain the rigorous solution^19–21^, while the physical insight has to invoke some other approaches for understanding the wave propagation characteristics. For example, The extraction method for determining the effective index and impedance from the scattering parameters of a finite slab of metamaterial normally incident by a plane wave was developed^22^. The S-parameter retrieval was employed to obtain the effective optical properties including permittivity and permeability of the fabricated zero index medium based on purely dielectric constituents^23^. The extraction of the effective medium properties (refractive index and impedance) of symmetric and asymmetric nanoparticle arrays with arbitrary geometry was developed and verified with analytical approach in the limitation of electrical small^24^. The dielectric permittivity tensor of the effective non-local medium with a periodic stack of graphene layers was developed for demonstrating its tunability from elliptic to hyperbolic dispersion with an external gate voltage^25^.

In this research, we focus on studying the physical mechanism of wave process involved in the so-called a series of mini photonic bandgaps reported in literature^19,26^. As far as a photonic bandgap is concerned, propagation constant against operating frequency (or wavelength) particularly in the stopband has to be carefully examined. Because an extremely large number of periods (unit cells) are considered here, the dispersion relation of wave propagation in the structure of infinite in extent can help the understanding of physical insight in wave mechanism. Furthermore, the Bloch impedance is essential to the impedance matching problem at the input/output interface considering a finite structure. Specifically, the effective refraction index and Bloch impedance were obtained based on Floquet-Bloch theory (periodic boundary condition). By solving the eigenvalue problem of the transfer matrix of a unit cell, the Bloch impedance and effective refractive index can be determined by the eigenvectors and eigenvalues, respectively. Consequently, the finite periodic structure can be modeled as an equivalent transmission line with effective (average) propagation constant and line (Bloch) impedance. The excellent agreement of the numerical results in the scattering analysis between transfer matrix method and Floquet-Bloch approach allows us to confidently interpret the periodic reflections using the effective refractive index and Bloch impedance. Additionally, the effect of chemical potential on the scattering properties and the equivalent transmission line parameters were also investigated intensively.

## Structure under consideration

**Figure 1:**
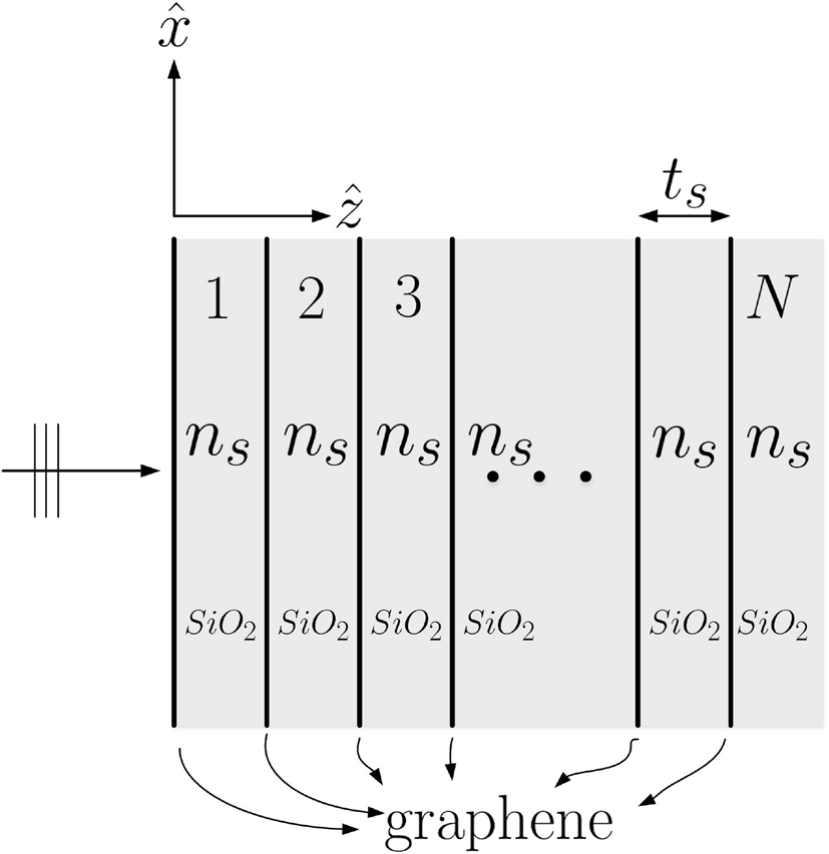
Structure configuration of a stratified graphene-dielectric metamaterial.

Figure 1 shows a stratified graphene-dielectric metamaterial. The structure is made of alternating graphene and silica ($SiO_2$) having thickness of $t_s$ and refractive index designated as $n_s$. The graphene sheet is assumed to be zero thickness with the graphene optical conductivity $\sigma _g$. The structure is composed of *N* periods (unit cells) each consisting of a graphene sheet and a $SiO_2$ slab. A plane electromagnetic wave is normally incident into the metamaterial. The graphene and $SiO_2$ slab are assumed to be infinite in extent along the *x*-*y* plane. Here, the input and output mediums both are set to be $SiO_2$ for reducing the reflection at input and output interfaces.

## Method of mathematical analysis

### Transfer matrix method (TMM)

Transfer matrix (or ABCD-matrix) method has been extensively employed in microwave and optical engineering^19,20^. Such a building block approach can efficiently calculate the scattering properties through cascade connection (matrix multiplication) of each input-output relation expressed in terms of a 2-by-2 matrix. Consider the multiple parallel dielectric and graphene layers in Fig. 1, the transmission-line analogy can be readily applied for describing the electric and magnetic fields within the dielectric layer^27,28^. At normal incidence, the tangential electric- and magnetic-fields in the uniform dielectric slab propagate along the *z*-axis with propagation constant $k_z^{(s)}=k_o n_s$; the wave impedance is simply the characteristic impedance of the medium $Z_s(=1/Y_s)=120\pi /n_s$, where $Y_s$ is the characteristic admittance. Moreover, the tangential electric and magnetic fields can be respectively written as $E_t(z)=V(z)$ and $H_t(z)=I(z)$, where the vector electric- and magnetic-fields are both on the *x*-*y* plane and perpendicular to each other; for example, $E_x$ and $H_y$ or $E_y$ and $H_x$. Additionally, *V*(*z*) and *I*(*z*) satisfy the transmission-line equations written as:

1 $$\begin{aligned} V(z)=A\exp (-jk_z^{(s)} z)+B\exp (+jk_z^{(s)} z), \end{aligned}$$

2 $$\begin{aligned} I(z)=Y_s \left[ A\exp (-jk_z^{(s)} z)-B\exp (+jk_z^{(s)} z) \right] \end{aligned}$$

Parameters *A* and *B* are two unknowns to be determined.

At the input interface of $z=t_s$, *A* and *B* can be expressed in terms of $V(t_s)$ and $I(t_s)$, that is, $A=\exp (+jk_z^{(s)} z)[V(t_s)+Z_s I(t_s)]/2$ and $B=\exp (-jk_z^{(s)} z)[V(t_s)-Z_s I(t_s)]/2$. By substituting *A* and *B* into Eqs. (1) and (2), *V*(*z*) and *I*(*z*) at $z=0$ can be denoted via use of $V(t_s)$ and $I(t_s)$, presented in the form of matrix equation given below:

3 $$\begin{aligned} \begin{bmatrix} V(0) \\ I(0) \end{bmatrix}={\mathbf {T}}_{SiO_2} \begin{bmatrix} V(t_s) \\ I(t_s) \end{bmatrix}, \end{aligned}$$

with the transfer matrix of the dielectric slab ($SiO_2$) written as:

4 $$\begin{aligned} {\mathbf {T}}_{SiO_2}=\begin{bmatrix} \cos k_z^{(s)}t_s &{} jZ_s \sin k_z^{(s)}t_s \\ jY_s \sin k_z^{(s)}t_s &{} \cos k_z^{(s)}t_s \end{bmatrix} . \end{aligned}$$

Additionally, consider a graphene sheet placed at the interface, $z=0$, between two regions denoted as (1) and (2) in $z<0$ and $z>0$, respectively. The boundary conditions^6^ of this zero thickness graphene sheet are ${\hat{z}}\times [{\mathbf {H}}^{(2)}_t(z=0^+)-{\mathbf {H}}^{(1)}_t(z=0^-)]=\sigma _g{\mathbf {E}}_t(z=0)$ and ${\mathbf {E}}^{(2)}_t(z=0^+)={\mathbf {E}}^{(1)}_t(z=0^-)$. Alternatively, we have $I^{(2)}(0^+)-I^{(1)}(0^-)=-\sigma _g V^{(1)}(0^-)$ and $V^{(1)}(0^-)= V^{(2)}(0^+)$. They can be expressed in term of the matrix equation written below:

5 $$\begin{aligned} \begin{bmatrix} V^{(1)}(0^-) \\ I^{(1)}(0^-) \end{bmatrix}={\mathbf {T}}_{gra} \begin{bmatrix} V^{(2)}(0^+) \\ I^{(2)}(0^+) \end{bmatrix} , \end{aligned}$$

with the transfer matrix of a graphene sheet written as:

6 $$\begin{aligned} {\mathbf {T}}_{gra}=\begin{bmatrix} 1 &{} 0 \\ \sigma _g &{} 1 \end{bmatrix} . \end{aligned}$$

Due to the continuous of tangential electric and magnetic fields at the interface between aforementioned two building blocks, the transfer matrix of the unit cell (period) is written as ${\mathbf {T}}_{cell}={\mathbf {T}}_{gra}{\mathbf {T}}_{SiO_2}$. Furthermore, the transfer matrix of a periodic structure consisting of *N* unit cells can be written as ${\mathbf {T}}=({\mathbf {T}}_{cell})^{N}$. Here, ${\mathbf {T}}$ is a 2-by-2 matrix. The transmittance (or insertion loss in microwave engineering) denoted as $S_{21}$ can be written as^29^:

7 $$\begin{aligned} S_{21}=\frac{2}{A+B/Z_o+C Z_i+D Z_i/Z_o} \end{aligned}$$

Additionally, the reflectance (or termed as return loss) is given as:

8 $$\begin{aligned} S_{11}=\frac{A+B/Z_o-C Z_i-D Z_i/Z_o}{A+B/Z_o+C Z_i+D Z_i/Z_o} \end{aligned}$$

where $Z_i$ and $Z_o$ are the input- and output-characteristic impedance in the input and output regions, respectively. Additionally, we have $A={\mathbf {T}}(1,1)$, $B={\mathbf {T}}(1,2)$, $C={\mathbf {T}}(2,1)$, and $D={\mathbf {T}}(2,2)$. Notably, parameter $S_{11}$ and $S_{21}$ are defined as the reflection- and transmission-coefficients; they, in general, are complex numbers. The reflected- and transmitted-power can then be obtained through $S_{11}$ and $S_{21}$^20^. Moreover, the reflectance and transmittance are determined by normalizing them with the incident power.

### Floquet-Bloch approach (FBA)

For an infinite periodic structure, the wave propagating characteristics can be understood from the property of its unit cell. By the Floquet-Bloch theory, the input-output relation of a unit cell satisfies

9 $$\begin{aligned} {\mathbf {T}}_{cell}{\mathbf {x}}=\chi {\mathbf {x}}, \end{aligned}$$

where ${\mathbf {x}}$ is a column vector composed of voltage and current amplitudes at the input end, and two eigenvalues $\chi =\exp (\pm j\kappa t_s)$. Parameter $\kappa =\beta -j\alpha$ is the effective propagation constant of the wave propagating through the unit cell. Namely, in such an infinite periodic medium, the wave can propagate in an “average” propagation constant $\kappa$. Therefore, the effective refractive index is defined as $n_{eff}=\kappa /k_o$. Notably, parameter $\kappa$ generally is a complex number due to that of the graphene conductivity $\sigma _g$.

Equation (9) is an eigenvalue problem. Having the given parameters in matrix ${\mathbf {T}}_{cell}$, the eigenvalue $\chi$ and eigenvector ${\mathbf {x}}$ can be readily determined. Furthermore, the Bloch impedance can be written as $Z_B={\mathbf {x}}(1)/{\mathbf {x}}(2)$. Notably, two eigenvectors will be obtained, the criterion for choosing the correct $Z_B$ is that its real part must be positive. Contrarily, $\chi =\exp (\pm j k_0 n_{eff} t_s)$ is a multiple-valued function of $n_{eff}$. Namely, $k_0 n_{eff} t_s+q2\pi$, where $q\in$ integer (branches) are also their solutions. More specifically, the real- and imaginary-parts of $n_{eff}=n_{eff}^{'}-j n_{eff}^{''}$ can be determined as follows.

10 $$\begin{aligned} n_{eff}^{''}=\pm \frac{\ln |\chi |}{2\pi }\frac{\lambda }{t_s} \end{aligned}$$

11 $$\begin{aligned} n_{eff}^{'}=\pm \frac{\phi _{\chi }}{2\pi }\frac{\lambda }{t_s}-q \frac{\lambda }{t_s}, \end{aligned}$$

where eigenvalue $\chi =|\chi |e^{j\phi _{\chi }}$ and $\lambda$ is the operating wavelength.

From Eqs. (10) and (11), we know that the imaginary part of $n_{eff}$ can be uniquely determined ($n_{eff}^{''} \ge 0$ for passive medium), while the real part of $n_{eff}$ accommodates multiple values. We have to point out that there is no approximation in the formulation of FBA. The obtained effective refractive index and Bloch impedance (even for the non-physical solutions of $\Re {n_{eff}}$) can be employed as transmission line parameters for evaluating the scattering properties of metamaterials having a large number of periods.

### Graphene optical conductivity

Graphene conductivity ($\sigma _g=\sigma _{intra}+\sigma _{inter}$), having a close-form expression for the condition $\mid \mu _c \mid$ $\gg$ $k_B T$, consists of both the intraband ($\sigma _{intra}$) and inter-band ($\sigma _{inter}$) terms:^6,30^

12 $$\begin{aligned} \sigma _{intra}(\omega )=\frac{-j e^2 |\mu _c|}{\pi \hslash ^2(\omega -j\gamma )} , \text{ and } \end{aligned}$$

13 $$\begin{aligned} \sigma _{inter}=\frac{e^2}{4\hslash } \left \{\frac{1}{2}+\frac{1}{\pi } \arctan \frac{\hslash (\omega -j\gamma )-2\mu _c}{2k_B T} +\frac{j}{2\pi }\ln \frac{[\hslash (\omega -j\gamma )+2\mu _c]^2}{[\hslash (\omega -j\gamma )-2\mu _c]^2+(2k_BT)^2} \right \} , \end{aligned}$$

where -*e* is the electron charge, $\hslash$ is the reduced Planck constant, $\gamma$ is a phenomenological carrier scattering rate ($\gamma =1/2\tau _c$, where $\tau _c$ is the carrier relaxation time), $\mu _c$ is the chemical potential, $k_B$ is Boltzmann’s constant, and *T* is the ambient temperature (assumed to be 300° K throughout this paper).

## Numerical results and discussions

Before the elaborate calculations, we have to first understand the graphene optical conductivity against frequency. Here, the normalized angular frequency $\Omega$ is defined as: $\Omega =\omega \hslash /2\mu _c^{(o)}$, where we have $\mu _c^{(o)}=0.35 eV$ throughout this paper. The silica thickness ($t_s$) equals to $\lambda _o/4$ (442.8007 nm), where $\lambda _o$ corresponds to the angular frequency of $\omega _o\hslash =2\mu _c^{(o)}$.

**Figure 2:**
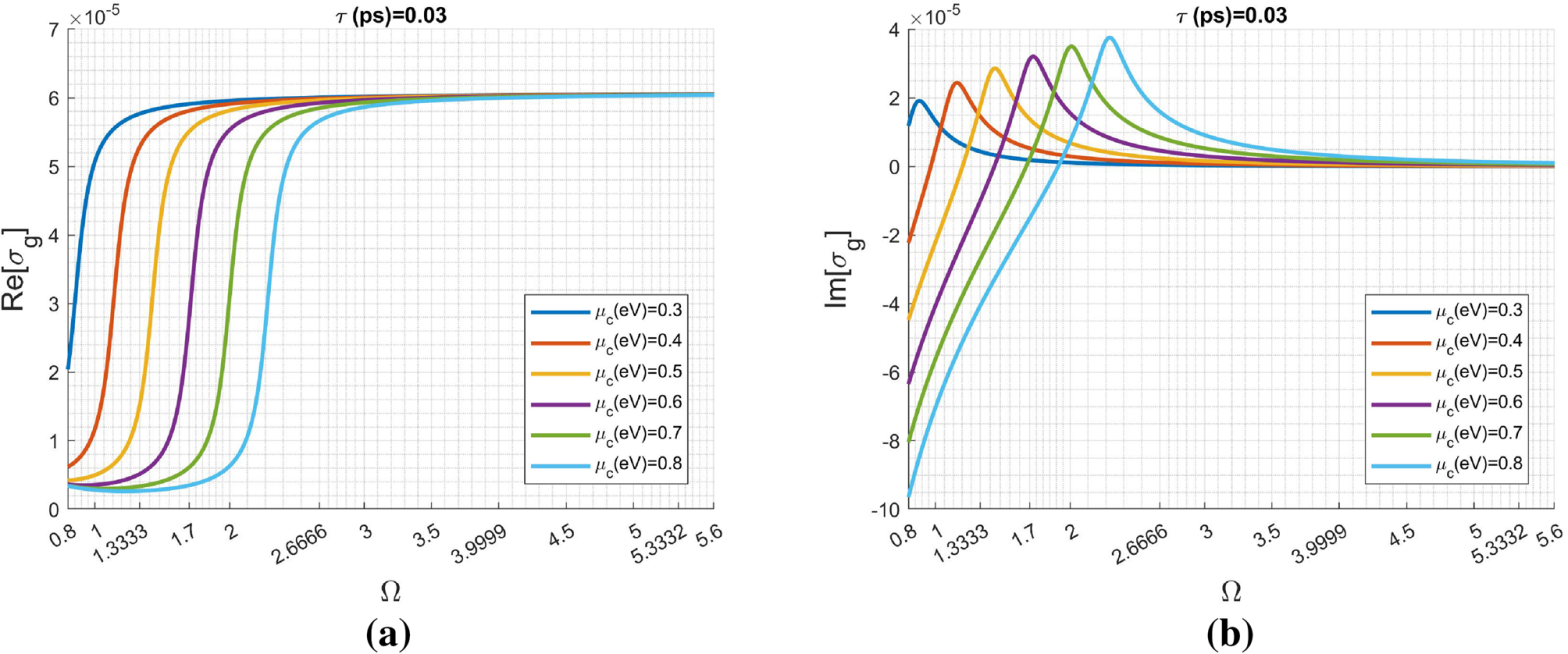
Graphene optical conductivity versus normalized angular frequency $\Omega =\hslash \omega /2\mu _c^{(o)}$ (where $\mu _c^{(o)}=0.35 \; \text{eV}$) for various chemical potential $\mu _c$: (**a**) real part and (**b**) imaginary part. The carrier relaxation time $\tau _c=0.03 \; \text{ps}$ and temperature of $T=$ 300° K.

Figure 2a,b respectively show the real- and imaginary-part of graphene optical conductivity due to the summation of Eqs. (12) and (13) against $\Omega$ for various chemical potential while having a fixed relaxation time $\tau _c=0.03 ps$ and temperature of $T=$ 300° K. Notably, the real- and imaginary-parts of $\sigma _g$ have significant changes with respect to the variation of $\mu _c$ in the low normalized frequency region, while they coincide to one another for high frequency region (for example, $\Omega > 5$).

**Figure 3:**
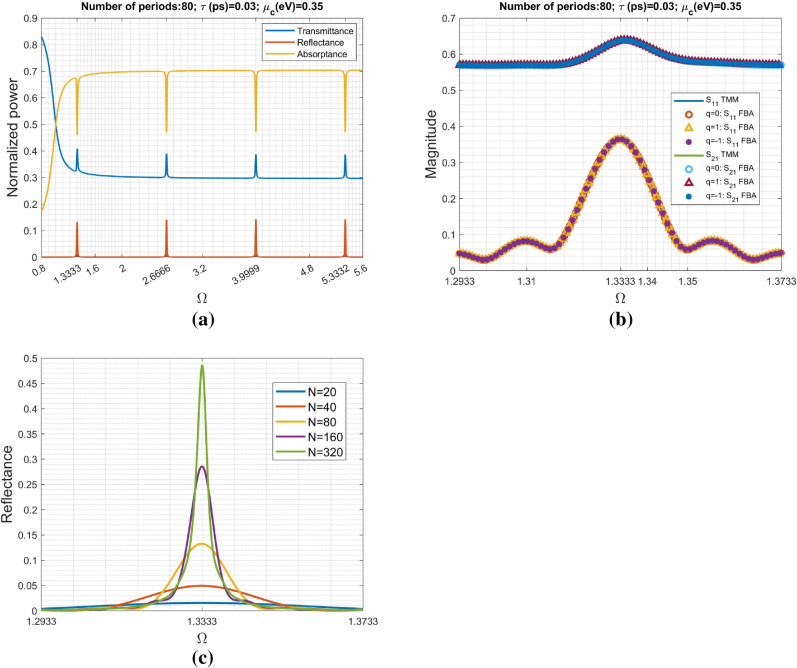
Scattering characteristics against normalized angular frequency ($\Omega$): (**a**) the power efficiency of transmittance, reflectance and absorptance (normalized to that of the incident power), and (**b**) scattering parameters against normalized frequency (around $\Omega =1.3333$) obtained through the approaches including transfer matrix method and Floquet-Bloch theory. The number of unit cells (periods) is 80. The graphene sheet has the following parameters: $\mu _c=0.35 \;\text{eV}$, $T=$ 300° K, and $\tau =0.03 \; \text{ps}$. The dielectric slab is $SiO_2$ having refractive index $n_s=1.5$ and thickness of 442.8007 nm. The input and output regions both are $SiO_2$ to improve the impedance matching at interfaces. Figure (**c**) shows the reflectance response around the first peak in (**a**) for various *N* (number of periods).

Figure 3a shows the scattering characteristics including transmittance, reflectance and absorptance against normalized angular frequency through the rigorous calculation by TMM. It is interesting to observe the frequency-selective reflection and transmission corresponding to the spikes. In addition to the strong reflection, the periodic absorption dips are also found at $\Omega =1.3333\;\text{m}$, where *m* is an integer starting from unity.

Moreover, the scattering parameters including $S_{21}$ and $S_{11}$ defined in Eqs. (7) and  (8) also were calculated via both approaches including TMM and FBA, particularly around the first peak at $\Omega =1.3333$, in Fig. 3b. Those symbol curves were obtained by FBA having different branches ($q=0$, $q=+1$, and $q=-1$). It is obvious to see the excellent agreement between the results obtained by the two methods. Although not shown here, the other branches including the other non-physical $Re[n_{eff}]$ ($Re[\cdot ]$ is referring the real part of a complex number) have also been examined and found consistent results of the scattering parameters compared with those obtained by TMM. Additionally, the periodic reflection has a bandwidth centered at around $\Omega =1.3333$. However, the reflection peak does not always coincides with $\Omega =1.3333$ for the other cases of $\mu _c$, as will become clear later on.

Figure 3c shows the reflectance response around the first reflection peak depicted in Fig. 3a. Apparently, the reflectance is increasing in accordance with the increase of *N* (number of periods). Specifically, their peak positions remain for all the cases with fewer or more periods. Although not shown here, the other reflection peaks in Fig. 3a also keep their positions and are independent of *N*. It reveals that those reflectance peaks are due to periodic nature of the structure under consideration.

Although multiple branches including non-physical solutions were obtained in the real part of effective refractive index due to multiple-valued problem, there is no ambiguity in determining the imaginary part of effective refractive index and the Bloch impedance. In fact, the reflection coefficient of a finite length metamaterial consisting of *N* unit cells is determined by $\Gamma =(Z_{in}-Z_s)/(Z_{in}+Z_s)$, where the input impedance can be written as follows.

14 $$\begin{aligned} Z_{in}=Z_B \frac{1+\Gamma _l e^{-j2k_o n_{eff} Nt_s}}{1-\Gamma _l e^{-j2k_o n_{eff} Nt_s}} \end{aligned}$$

where $$\Gamma _l=(Z_s-Z_B)/(Z_s+Z_B)$$.

Notably, $e^{-j2k_o n_{eff} Nt_s}=\chi ^{2N}$ and $\chi$ is the eigenvalue in Eq. (9); there is no ambiguity in determining $\chi$. As a consequence, the equivalent transmission line using $\chi$ and $Z_B$ can uniquely determine the scattering parameters and no need to consider the multiple-valued problem.

**Figure 4:**
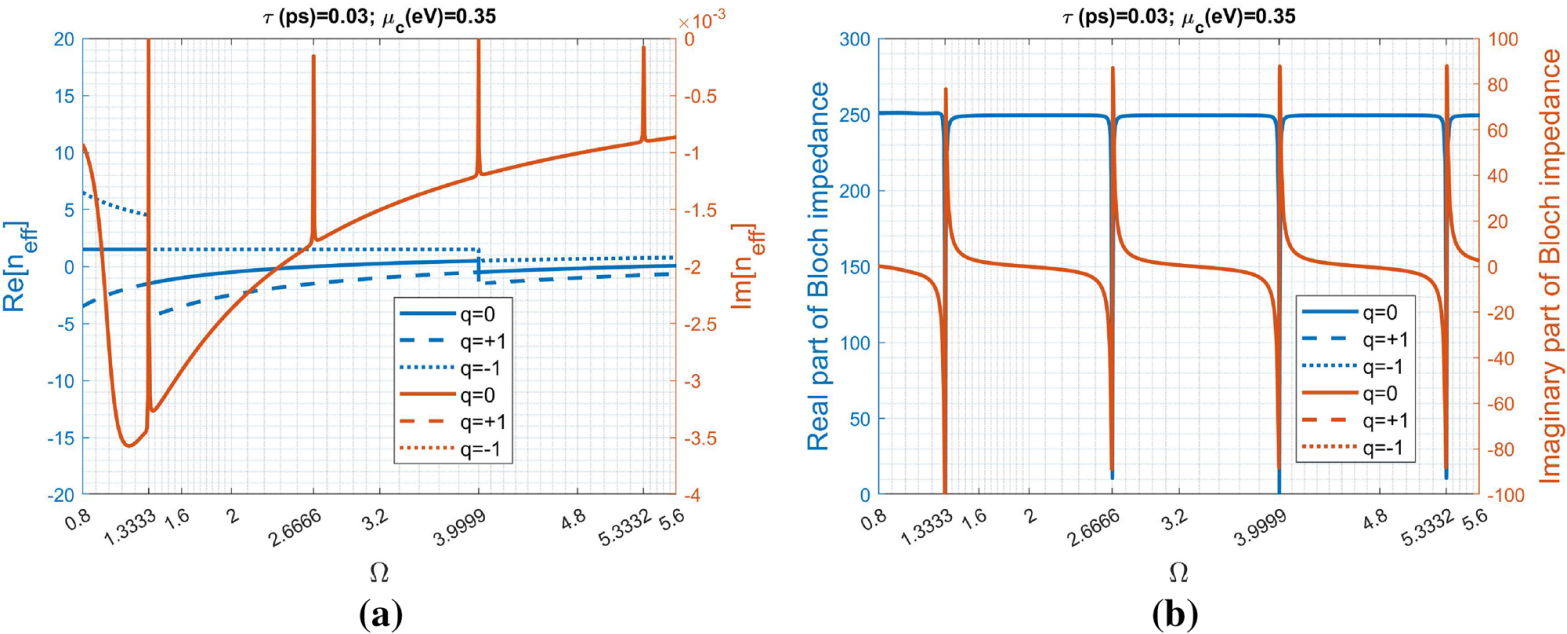
Effective refractive index and Bloch impedance against normalized angular frequency of the global view: (**a**) the real- and imaginary-parts of the effective refractive index ($n_{eff}$), and (**b**) the real- and imaginary-parts of the Bloch impedance ($Z_B$).

Figure 4a,b individually depict the effective refractive index and Bloch impedance versus normalized angular frequency in the same range shown in Fig.  3a. It is obvious to see that the frequencies with transmission/reflection spikes coincide with those having negligible $Im[n_{eff}]$ ($Im[\cdot ]$ means the imaginary part of a complex number). Additionally, those peaks with vanishing $Im[n_{eff}]$ occur at $\Omega =1.3333\;\text{m}$, where *m* is ranging from 1 to 4. Because of multiple-valued function of $Re[n_{eff}]$, all the integer *q* should be taken into account. However, in this figure only the three branches: the blue solid, dashed and dotted curves individually corresponding to branches of $q=0$, $q=+1$ and $q=-1$, are plotted. On the other hand, their imaginary part share the same distribution. In the low frequency region with $\Omega<< 1$ ($t_s<< \lambda$), the branches of $q\ne 0$ are away from the principal branch of $q=0$; there is no ambiguity in selection of the correct branch (*q*). However, it creates difficulty in unambiguously determining the correct branch when $\Omega > 1$ since the $Re[n_{eff}]$ of the three branches gradually lie quit close.

Additionally, the Bloch impedance versus normalized angular frequency is also plotted and shown in Fig.  4b. There is no ambiguity in determining the Bloch impedance; thus all the cases of different *q* coincide with one another. Significantly, the Bloch impedance at $\Omega =1.3333$, $\Omega =2.6666$, $\Omega =3.9999$, and $\Omega =5.3332$ are $Z_B=4.381\times 10^{-5}-j5.052\times 10^{-5}$, $Z_B=3.692\times 10^{-5}+j3.650\times 10^{-5}$, $Z_B=6.669\times 10^{-5}-j6.691\times 10^{-5}$, and $Z_B=9.155\times 10^{-5}-j9.169\times 10^{-5}$, respectively.

### Determine the frequencies around periodic reflections

To explain the vanishing imaginary part of $n_{eff}$, we return to the eigenvalue problem in Eq. (9). Its alternative expression (the characteristic equation of matrix $\mathbf {T_{cell}})$ can be written below.

15 $$\begin{aligned} \cos (k_o n_{eff} t_s)=\cos (k_o t_s n_s)+j\frac{\sigma _g Z_s}{2}\sin (k_o t_s n_s) \end{aligned}$$

Equation (15) is also termed as the dispersion relation of wave propagating in the 1D periodic medium. The effective refractive index ($n_{eff}$) can be resolved once the parameters including $k_o$, $n_s$ and graphene conductivity ($\sigma _g$) are given. We first consider the condition of $k_o t_s n_s =m\pi$, where *m* is an integer excluding zero, enabling $\sin (k_o t_s n_s)=0$. Consequently, we have $\cos (k_o n_{eff} t_s)=\pm 1$; $n_{eff}$ is a pure real number. It means that the wave propagating in the medium at this frequency has no attenuation.

Additionally, the zero $Im[n_{eff}]$ at $k_o t_s n_s =m\pi$ corresponds to the angular frequency $\omega _m=C_o m \pi /n_s t_s$, where $C_o$ is the speed of light. The normalized angular frequency is $\Omega _m=m \hslash C_o \pi /2 n_s t_s \mu _c^{(o)}$. Substitution of $\mu _c^{(o)}=0.35 \; \text{eV}$, $n_s=1.5$ and $t_s=442.8007\;\text{nm}$ into $\Omega _m$, we obtain $\Omega _m=1.3333\;\text{m}$.

Parameter $Re[n_{eff}]$ can accommodate multiple values; however, the eigenvector to Eq. (9) is uniquely determined. Notably, the eigenvectors at the condition of vanishing $Im[n_{eff}]$ are repeated eigenvalues problem and should be carefully evaluated. Specifically, the Bloch impedance has very small real and imaginary parts. Notably, the effective refractive index $n_{eff}$ has negligible imaginary part at the normalized frequencies equal to $\Omega _m$. Nevertheless, the non-zero complex Bloch impedance enables the power absorption by the structure due to the presence of graphene sheets.

**Figure 5:**
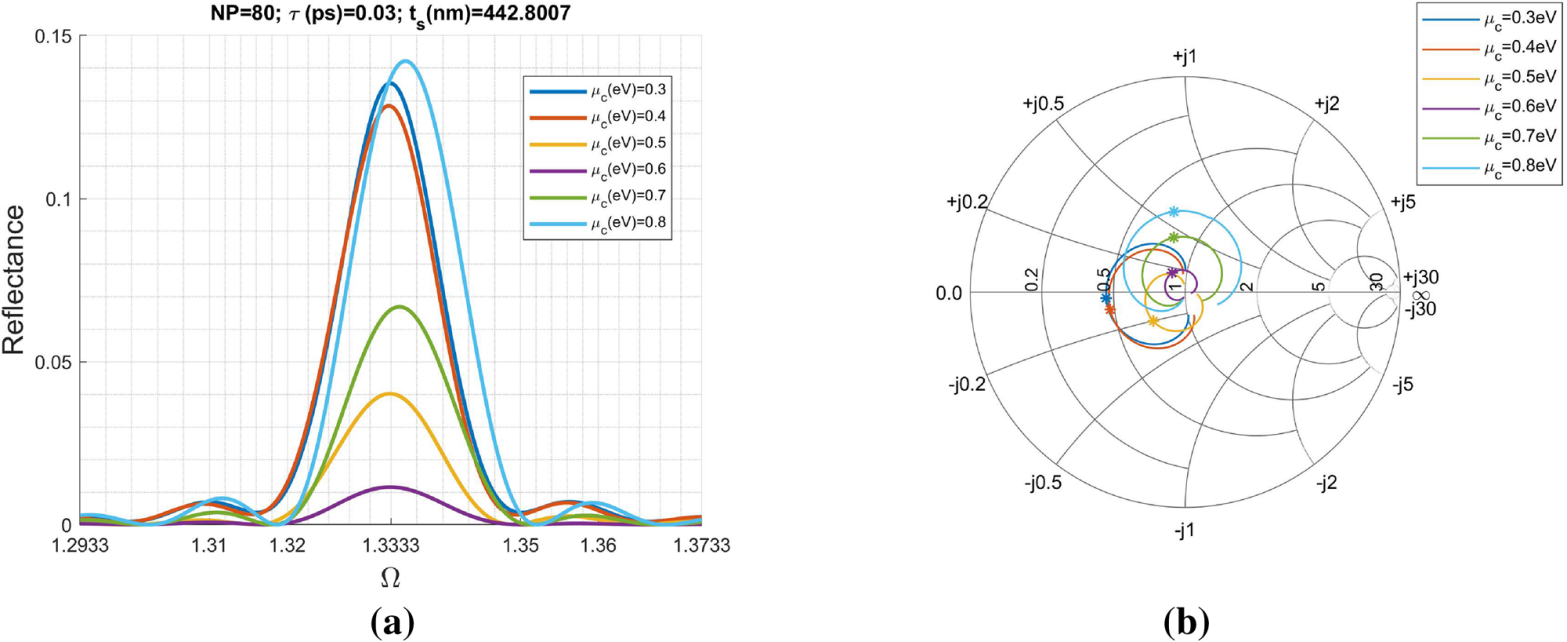
Reflectance and input impedance for various chemical potential ($\mu _c$): (**a**) reflectance is plotted as a function of normalized angular frequency ($\Omega$) and, (**b**) the distribution of input impedance (normalized to $Z_s$) around each reflectance peak is individually plotted in the Smith Chart. The number of unit cells (periods) is 80. The graphene sheet has the following parameters: $T=$ 300° K, and $\tau =0.03 \; \text{ps}$. The dielectric slab is $SiO_2$ having refractive index $n_s=1.5$ and thickness of 442.8007 nm. The input and output regions both are $SiO_2$.

### Effect of chemical potential on reflection characteristics

In Fig. 5a, reflectance versus normalized angular frequency were demonstrated. The first peak at $\Omega =1.3333$ corresponds to $\lambda =1328.402 \; \text{nm}$ while the size of unit cell is $t_s=442.8007 \; \text{nm}$. Therefore, it is in the long-wavelength limit. There is no ambiguity in determining the $Re[n_{eff}]$. To see the effect of chemical potential ($\mu _c$) on the reflection properties, we progressively change $\mu _c$ from 0.3*eV* to 0.8 eV with a step of 0.1 eV. Incidentally, we also have evaluated them at $\mu _c=0.1\;\text{eV}$ and $\mu _c=0.2\;\text{eV}$; however, the results were not shown here because their difference with that of $\mu _c=0.3\;\text{eV}$ is insignificant.

Returning to Fig. 5a, the obvious reflection occurs in the region roughly between $\Omega =1.32$ and $\Omega =1.35$. Hereafter, this region is referred to as “A (anomalous)-region”. Outside the A-region, reflectance is inconsiderable. On the other hand, the increase in $\mu _c$ gradually moves the peak position away from $\Omega =1.3333$.

It is obvious to see the strength of reflectance peak varies irregularly with the increase in chemical potential. Because the reflectance is determined by $\Gamma =(z_{in}-1)/(z_{in}+1)$, with $z_{in}=Z_{in}/Z_s$ the input impedance normalized to $Z_s$, the impedance match between $Z_{in}$ and $Z_s$ dominates the reflectance. As will become clear later on, both $n_{eff}$ and $Z_B$ are frequency dependence and have drastic variations in the A-region; specifically, their relation to $\mu _c$ are irregular. Consequently, it is hard to explicitly define the relationship between $\mu _c$ and reflectance. However, the input impedance in Eq. (14) can reveal us this information. Figure 5b shows the distribution of normalized input impedance ($z_{in}$) against $\Omega$. The locus of each case is drawn in the Smith Chart that is commonly used in microwave engineering. The center is at the point of $1+j0$ representing normalized impedance equal to unity (perfect match without reflection). The reflectance peak of each case shown in Fig. 5a is marked in Fig. 5b respectively. Moreover, the distance between the point on a locus and the center can be employed to evaluate the impedance matching (or level of reflectance). From Fig. 5b, it is obvious to see that the relationship of $d(0.6\;\text{eV})<d(0.5\;\text{eV})<d(0.7\;\text{eV})<d(0.4\;\text{eV})<d(0.3\;\text{eV})<d(0.8\;\text{eV})$, where $d(\mu _c)$ is the distance between peak denoted by star symbol and center for the chemical potential given within the round brackets. Apparently, the smaller the distance *d*, the better impedance match and lower reflectance achieved, shown in Fig. 5a.

**Figure 6:**
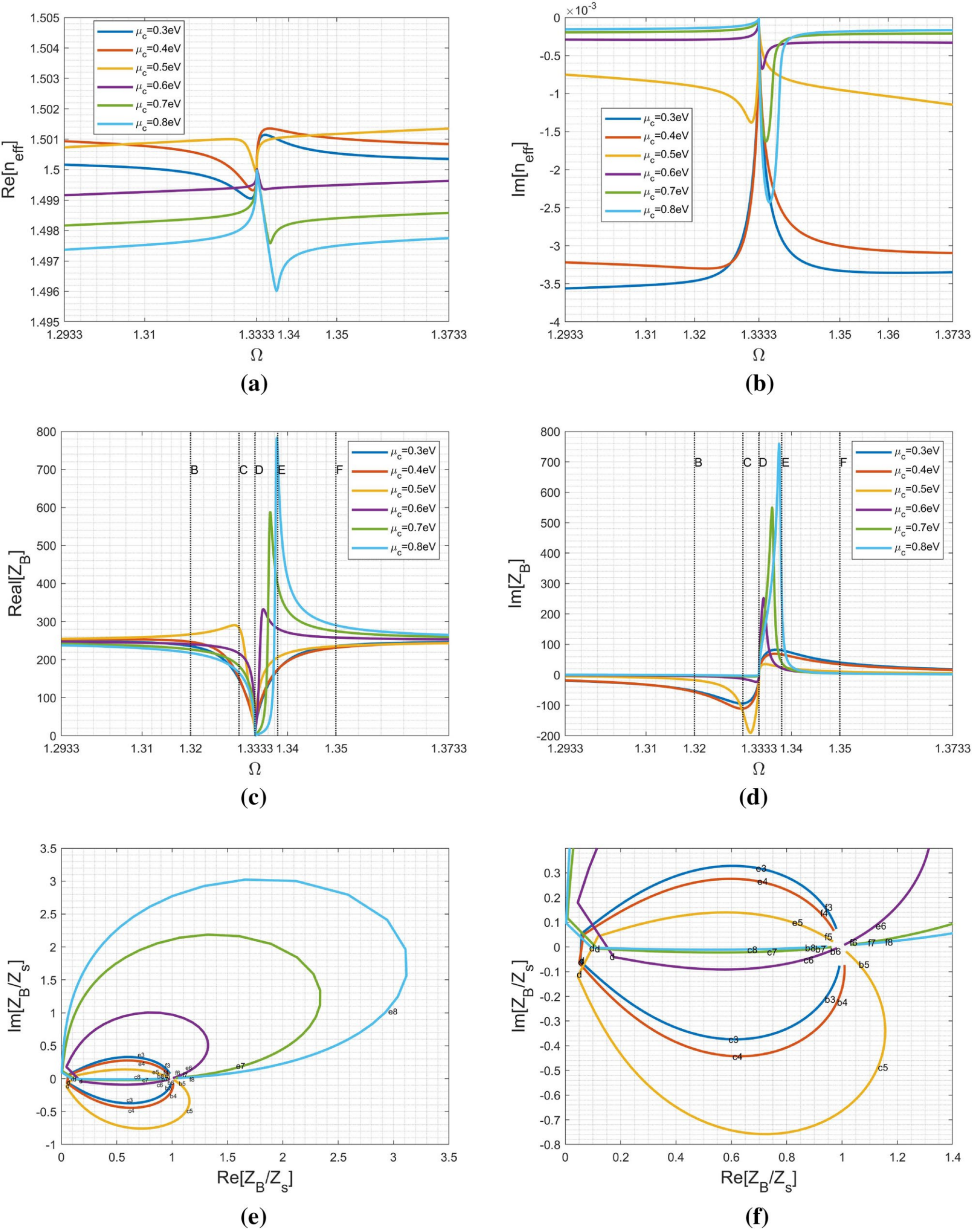
Effective refractive index and Bloch impedance against normalized angular frequency at around the first reflection peak of $\Omega =1.3333$: (**a**) real part of the effective refractive index, (**b**) imaginary part of the effective refractive index, (**c**) real part of the Bloch impedance, (**d**) imaginary part of the Bloch impedance, (**e**) the normalized Bloch impedance ($Z_B/Z_s$) plotted in a 2D plane, and (**f**) enlarged view of (**e**) around $\Omega =1.3333$.

### Effect of chemical potential on the effective refractive index and Bloch impedance

To explain the obvious reflection in the vicinity of $\Omega =1.3333$ shown in Fig. 5a, the effective refractive index and Bloch impedance were calculated for various chemical potential given in the aforementioned examples. Figure 6a,b show the variation of $n_{eff}$ against the normalized frequency. As depicted in Fig. 6a, $n_{eff}^{'}$ approaches the index of surrounding medium ($n_s$) for all $\mu _c$ in the full band except for the abrupt change in slope found around normalized frequency of 1.3333, may causing the change in their group velocity.

The imaginary part of refractive index ($n_{eff}^{''}$) allows us to know attenuation of Floquet-Bloch mode propagating in an infinite periodic medium. In Fig. 6b, all the cases experience zero attenuation at $\Omega =1.3333$. For the case of $\mu _c=0.3$ and $\mu _c=0.4$, it behaves like a band-pass filter having a narrow pass band around $\Omega =1.3333$. Contrarily, the cases of $\mu _c=0.7$ and $\mu _c=0.8$ encounter apparent attenuation (or reflection for the incident wave) inside the bump shape region starting from $\Omega =1.3333$. Additionally, the cases of $\mu _c=0.5$ and $\mu _c=0.6$ have a small fluctuation in their propagation. Notably, the aforementioned properties are subject to an infinite medium without considering the input/output interface. Their behavior are very different from the result shown in Fig. 5a. As a consequence, we know that the only parameter $n_{eff}$ cannot afford to explain reflectance response.

As is well known in a 1D periodic medium, the stop-band is due to the coherent reflection from each unit cell. When we evaluate the dispersion relation of the 1D wave propagating in an infinite periodic medium, the propagation constant is a complex number ($\kappa =\beta -j\alpha$) in the stop-band regions, while it is a real number in the pass-band regions. Contrarily, when inspecting $n_{eff}$ in Fig.6a,b especially in the A-region, we found that such a behavior can not be classified simply as a stopband. Additionally, the abrupt change $n_{eff}$ around $\Omega =1.3333$ is due to structure dispersion, while the variation of $n_{eff}(\Omega )$ with respect to $\mu _c$ is attributed to material dispersion.

The reflection is mainly due to the mismatch between the input impedance of a metamaterial and the wave impedance of the surrounding medium. First of all, outside the A-region, all the cases in Fig. 6c,d generally approach the wave impedance in $SiO_2$ ($Z_s=251 \Omega$). This explains the small reflection outside the A-region. Contrarily, it is apparent to see drastic changes in the real- and imaginary-parts of Bloch impedance within the A-region. Specifically, the two cases of $\mu _c=0.7\;\text{eV}$ and $\mu _c=0.8\;\text{eV}$ exhibit complex Bloch impedance within a very narrow bandwidth starting from $\Omega =1.3333$ to around $\Omega =1.342$ while the others are complex numbers in the A-region. Furthermore, all the cases have extremely low Bloch impedance at $\Omega =1.3333$; however, its does not mean at all the location of peak reflection. In fact, it is not easy to precisely predict the peak position unless to calculate the input impedance looking into the metamaterial. Notably, both frequency-dependent $n_{eff}(\Omega )$ and $Z_B(\Omega )$ are essential for the calculation of input impedance via the formula given below:

16 $$\begin{aligned} Z_{in}=Z_B\frac{Z_s+jZ_B\tan (k_on_{eff}Nt_s)}{Z_B+jZ_s\tan (k_on_{eff}Nt_s)}. \end{aligned}$$

Nevertheless, from Fig. 5a, we found that the reflection peaks for all the cases are within the A-region and in the vicinity of $\Omega =1.3333$.

To facilitate the understanding for variation of Bloch impedance, shown in Fig. 6c,d, the complex impedance is normalized to $Z_s$ and redrawn in a 2D plot shown in Fig. 6e and a zoom in view around (1, 0) in Fig. 6f. The 2D curves can be distinguished by their colors corresponding to the chemical potential given in the legend of Fig. 6c,d. Moreover, the alphabetical letters attached to each loop correspond to the normalized frequency points ($\Omega$) labeled in the A-region. The index after the alphabetical letter stands for the value of chemical potential; for example, *c*7 means the case of $\mu _c=0.7\;\text{eV}$ at point C. This allows us to trace the variation of a normalized Bloch impedance in a complex plane. Each loop moves in a clockwise direction with its starting and stopping points locating around the point matching to the surrounding medium at (1, 0). At the point d ($\Omega =1.3333$), all the cases having low Bloch impedance distributed near the point of (0, 0). Since the loop size represents the level of impedance variation in the frequency band under consideration, we may conclude that Bloch impedance variation increases in accordance with the increase in chemical potential.

Although not shown here, the other frequencies with strong reflections in Fig. 3a also have drastic variations in the real- and imaginary-parts of Bloch impedance. Specifically, their distributions (both $n_{eff}(\Omega )$ and $Z_B(\Omega )$) are very similar for various $\mu _c$, in particular for $\Omega =3.9999$ and $\Omega =5.3332$, because their $\sigma _g(\Omega )$ almost coincide to one another for various $\mu _c$ shown in Fig. 2a,b.

**Figure 7:**
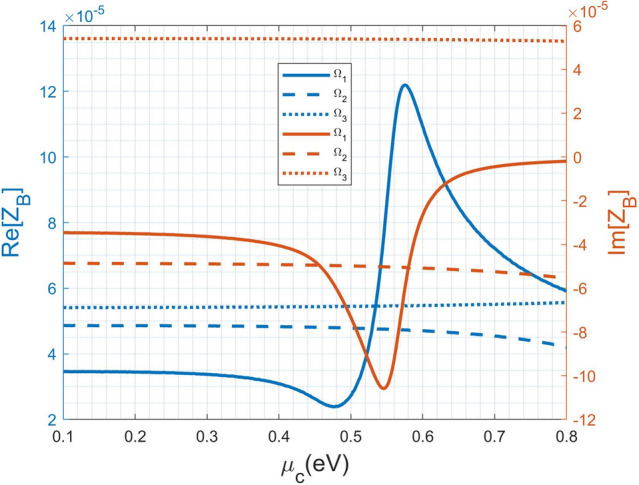
Variation of the Bloch impedance ($Z_B$) against chemical potential ($\mu _c$) for the first three modes with normalized frequencies $\Omega _1=1.3333$, $\Omega _2=2.66666$, and $\Omega _3=3.99999$, respectively.

In Fig. 7, we change the chemical potential to see its influence on the Bloch impedance. The first three normalized frequencies: $\Omega _1=1.3333$, $\Omega _2=2.6666$, and $\Omega _3=3.9999$ are considered. Return to Fig. 2a, in the vicinity of $\Omega _1=1.3333$ $Re[\sigma _g]$ has insignificant change for $\mu _c$ ranging from 0.1 to 0.3 eV. Big changes occur at the three cases including 0.4 eV, 0.5 eV, and 0.6 eV. The difference between the cases of 0.7 eV and 0.8 eV is inconsiderable. The aforementioned trend in the change of $Re[\sigma _g]$ also reflects the change in real- and imaginary-parts of $Z_B$. Contrarily, for the cases of $\Omega _2=2.6666$ and $\Omega _2=3.9999$, their variations on $\sigma _g$ due to various $\mu _c$ are not obvious shown in Fig. 2a,b. This is the reason why their Bloch impedance have insignificant change depicted in Fig. 7. Alternatively, the Bloch impedance (at the condition of $k_o n_s t_s=m\pi$) is determined by the eigenvector of the matrix ${\mathbf {T}}_{gra}$ in Eq. (6), which merely depends on graphene optical conductivity $\sigma _g$.

**Figure 8:**
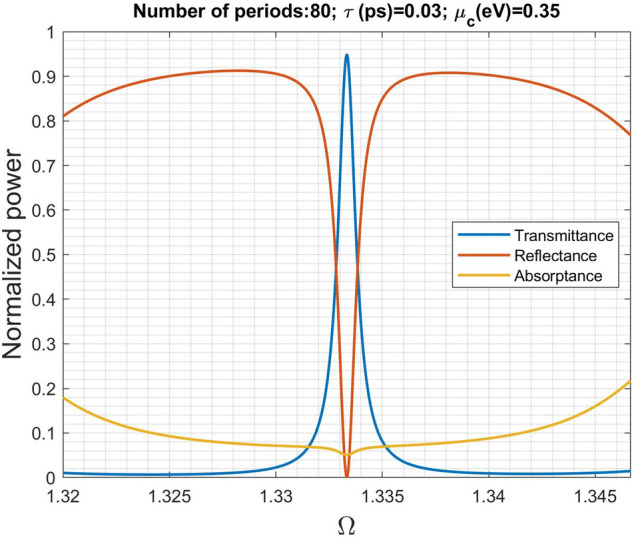
The scattering characteristics of the same 1D metamaterial but with the wave impedance equal to $11.922\Omega$ in the input and output region; the original one is $251\Omega$.

To demonstrate that impedance matching affects the scattering characteristic, we further reduce the wave impedance in the input and output regions from $251\Omega$ to $11.922\Omega$ (corresponding to $n_s=\sqrt{1000}$, the unnaturally high refractive index can be realized using metamaterials^31^) and carry out the scattering analysis. Notably, the structure parameters of the graphene metamaterial remains the same as in Fig. 3b. It is obvious to see in Fig. 8, the transmittance is greatly improved at around $\Omega _1=1.3333$ due to impedance match, while the impedance mismatch is enhanced outside the region. The structure turns to become periodic transmissions. This simulation reveals that the Bloch impedance plays an important role in scattering process.

## Conclusion

The Floquet-Bloch approach was employed to determine the effective refractive index and Bloch impedance of a metamaterial made of a stratified graphene-dielectric structure. Although multiple branches including non-physical solutions were obtained in the real part of effective refractive index due to multiple-valued problem, there is no ambiguity in determining the imaginary part of effective refractive index and the Bloch impedance. From the numerical results we confirm that FBA can correctly predict the scattering characteristics far beyond the long-wavelength limit.

Through this research, we found that the periodic reflections of a stratified graphene-dielectric metamaterial take place around the frequencies of $k_o n_s t_s=m\pi$ due to structure dispersion. Moreover, the strong fluctuations in the effective refractive index and Bloch impedance attribute to the material dispersion that can be altered by tuning the chemical potential. Additionally, the remarkable variations in both effective refractive index and Bloch impedance cause the drastic variation in input impedance, thereby enabling the occurrence of obvious reflection rather than due to photonic bandgap. Specifically, a Floquet-Bloch mode (state) having pure real effective refractive index and extremely low Bloch impedance was found to exist within the frequency bands of periodic reflections. Moreover, the effect of chemical potential on the reflection properties and equivalent transmission line parameters including refractive index and Bloch impedance have also been intensively studied.
